# Feasibility of Automated Volumetric Assessment of Large Hepatocellular Carcinomas' Responses to Transarterial Chemoembolization

**DOI:** 10.3389/fonc.2020.00572

**Published:** 2020-05-07

**Authors:** Ahmed W. Moawad, David Fuentes, Ahmed M. Khalaf, Katherine J. Blair, Janio Szklaruk, Aliya Qayyum, John D. Hazle, Khaled M. Elsayes

**Affiliations:** ^1^Imaging Physics Department, University of Texas MD Anderson Cancer Center, Houston, TX, United States; ^2^Diagnostic Radiology Department, University of Texas MD Anderson Cancer Center, Houston, TX, United States

**Keywords:** volumetric RECIST, hepatocellular carcinoma, TACE, automated segmentation, tumor response

## Abstract

**Background:** Hepatocellular carcinoma (HCC) is the most common liver malignancy and the leading cause of death in patients with cirrhosis. Various treatments for HCC are available, including transarterial chemoembolization (TACE), which is the commonest intervention performed in HCC. Radiologic tumor response following TACE is an important prognostic factor for patients with HCC. We hypothesized that, for large HCC tumors, assessment of treatment response made with automated volumetric response evaluation criteria in solid tumors (RECIST) might correlate with the assessment made with the more time- and labor-intensive unidimensional modified RECIST (mRECIST) and manual volumetric RECIST (M-vRECIST) criteria. Accordingly, we undertook this retrospective study to compare automated volumetric RECIST (A-vRECIST) with M-vRECIST and mRESIST for the assessment of large HCC tumors' responses to TACE.

**Methods:**We selected 42 pairs of contrast-enhanced computed tomography (CT) images of large HCCs. Images were taken before and after TACE, and in each of the images, the HCC was segmented using both a manual contouring tool and a convolutional neural network. Three experienced radiologists assessed tumor response to TACE using mRECIST criteria. The intra-class correlation coefficient was used to assess inter-reader reliability in the mRECIST measurements, while the Pearson correlation coefficient was used to assess correlation between the volumetric and mRECIST measurements.

**Results:**Volumetric tumor assessment using automated and manual segmentation tools showed good correlation with mRECIST measurements. For A-vRECIST and M-vRECIST, respectively, *r* = 0.597 vs. 0.622 in the baseline studies; 0.648 vs. 0.748 in the follow-up studies; and 0.774 vs. 0.766 in the response assessment (*P* < 0.001 for all). The A-vRECIST evaluation showed high correlation with the M-vRECIST evaluation (*r* = 0.967, 0.937, and 0.826 in baseline studies, follow-up studies, and response assessment, respectively, *P* < 0.001 for all).

**Conclusion:**Volumetric RECIST measurements are likely to provide an early marker for TACE monitoring, and automated measurements made with a convolutional neural network may be good substitutes for manual volumetric measurements.

## Introduction

Hepatocellular carcinoma (HCC) is the most common liver malignancy and the leading cause of death in patients with cirrhosis. Despite advances in various treatment modalities over the past several years, the prognosis for HCC remains poor, with 5-year overall survival ranging from 24 to 41% (1, 2). Efforts have been made to improve early detection of HCC by the performance of frequent screening in high-risk populations. However, most cases are still diagnosed at intermediate to advanced stages (3). These patients are not candidates for curative therapies, such as surgical resection or liver transplant. As a result, treatment options for this patient population are limited to loco-regional treatments, including local radiofrequency ablation, radio and chemoembolization, and systemic chemotherapy with Sorafenib (4–6).

In the United States, transarterial chemoembolization (TACE) is the most common intervention for HCC (7). It is the standard of care for patients with intermediate-stage HCC (according to Barcelona clinic liver cancer (BCLC) staging, whether it is large tumor or multi-nodular). In addition, it may be used in advanced HCC prior to systemic therapy or as a bridging therapy prior to surgery (8). There is evidence that repeat TACE may also be beneficial in patients with advanced HCC (9–11). Radiologic tumor response following initial TACE has been shown to be an important prognostic factor for patients with HCC. Baseline imaging is usually obtained 2–3 weeks before therapy and follow-up imaging is performed 4–6 weeks after therapy. The most commonly used criteria for tumor response following HCC is mRECIST (1-dimension) or EASL (2-dimensions).

The recent attempts to improve the accuracy of radiologic response criteria to predict overall survival and Progression-free survival have focused on using quantitative volumetric analysis. This has resulted in the development of the volumetric RECIST (vRECIST) and quantitative EASL (qEASL) methods with better results in predicting patient's outcome than the currently used mRECIST (12–15).

The volumetric assessment of tumor response depends on manual segmentation of tumor and needs contouring of the lesion in every single slice of the study. This process is time consuming and tedious. The manual volumetric assessment show high inter- and intra-observer variability which make it impractical in daily practice (16–18). On the other hand, automated volumetric segmentation has the potential to reduce time for this process and make volumetric assessment of tumors more practical, with lower inter and intra-observer variability than both mRECIST and manual volumetric assessment (12, 18, 19).

Convolutional neural network (CNN) shows promise to achieve automated segmentation of liver and liver masses. These are, however, computationally demanding (12, 13). CNN in tumor segmentation has been found to be more accurate and closer to the manual volumetric segmentation in larger tumors, with far lower accuracy in smaller liver masses (14, 15).

The purpose of this study is to assess the feasibility of volumetric assessment of pre- and post-TACE HCC using fully automated segmentation and to evaluate the correlation of automated volumetric assessment with both manual volumetric assessment and mRECIST measurements.

## Materials and Methods

### Study Cohort

This is a retrospective, single-institution, IRB approved study. This study included patients with large HCC tumors (≥5 cm) diagnosed and treated at our institution between November 2002 and June 2012. Patients were included in the study if (1) they had undergone TACE as their sole first-line or initial bridging therapy; (2) their medical records included multiphasic, contrast-enhanced CT images that were obtained at baseline and that included no image artifacts (e.g., surgical clips); and (3) their tumor was diagnosed as tumor-node-metastasis (TNM) stage III or IV HCC based on the American Joint Committee on Cancer (AJCC). Although there are numerous HCC scoring systems that incorporate liver functional reserve, patient performance, and gross tumor characteristics (e.g., size, vascular invasion, number of lesions), we chose the TNM staging system because it is the only HCC staging system that considers tumor characteristics (including size) without taking any other factor into consideration (20, 21).

### TACE

Briefly, TACE is delivery method of chemotherapy delivery to the tumor through its feeding arteries using trans-arterial approach. The hepatic artery was selected and injected with chemotherapy by super selection of the feeding vasculature using advanced micro-catheters. There are two main chemotherapeutic regimens that may be delivered. In conventional TACE, a mixture of radio-opaque ethiodized emulsion oil (Lipiodol; Guerbet, Villepinte, France) and doxorubicin or cisplatin was injected followed by embolization of the feeding vessels. While in TACE with drug eluting beads (DEB-TACE), a mixture of micro-sphere particles, doxorubicin and soluble non-ionic contrast was injected (22).

Our patients received one of the following chemotherapeutic regimens (Drug regimen details are missed from 2 cases): (1) embolic microsphere beads (Biocompatibles, UK) loaded with doxorubicin (DEBDOX; drug-eluting bead doxorubicin) (15 lesions), (2) cisplatin, doxorubicin, and mitomycin C (22 lesions), or (3) cisplatin and mitomycin C only (3 lesions) (such information is missed from 2 cases).

### CT Imaging Technique

All patients underwent dynamic, contrast-enhanced CT scans of the abdomen on 4-, 16-, or 64-slice multidetector CT LightSpeed scanners (GE Healthcare, Chicago, IL) pre-TACE and post-TACE. Liver protocol was used in all studies (the arterial, porto-venous, and delayed phases were captured 17, 60 s, and ~5 min, respectively, after peak enhancement of the descending aorta). Injection was done with an automated contrast injector using a bolus tracking technique and an injection rate of about 3–5 mL/s. The image reconstruction thicknesses were 2.5 mm and 5 mm.

### Assessment of Tumor Response

**Figure 1:**
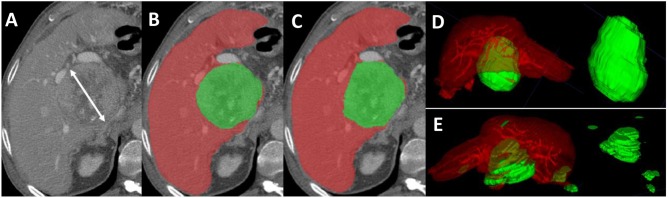
Axial sections from a pre-TACE abdominal CT scan of a 64-year old male patient with advanced HCC. **(A)** Shows an 8.5 cm lesion measured with mRECIST criteria. **(B)** Shows the manual segmentation of the hepatic parenchyma (red) and the HCC tissue (green); the tumor volume, measured with M-vRECIST, was 377.25 cm^3^. **(C)** Shows the automated segmentation of the parenchyma (red) and HCC tissue (green); the tumor volume measured with A-vRECIST was 187.9 cm^3^. **(D,E)** Show the 3-dimensional voxel rendering according to M-vRECIST and A-vRECIST, respectively. A-vRECIST, automated volumetric response evaluation criteria in solid tumors; CT, computed tomography; HCC, hepatocellular carcinoma; mRECIST, modified response evaluation criteria in solid tumors; M-vRECIST, manual volumetric response evaluation criteria in solid tumors; RECIST, response evaluation criteria in solid tumors; TACE, transarterial chemo embolization.

Tumor TACE response was assessed using mRECIST, M-vRECIST, and A-vRECIST (Figure 1). Three different board certified radiologists (KE, JS, and AQ), each with more than 20 years of experience in abdominal imaging, independently measured tumors using mRECIST criteria. The changes in measurements between the follow-up and baseline CT scans were reported, and tumor viability and enhancement in the late arterial phase were taken into consideration. Volume assessment using M-vRECIST and A-vRECIST at baseline and follow-up studies was also done. The Convert3D medical image processing tool was used to extract the segmentation volumes according to the voxel extensions (23).

### Tumor Segmentation

The porto-venous phase of CT (both baseline and follow-up) were used to simplify lesion assessment, they were exported in DICOM format from our institution's picture archiving and communication system to a separate research server. Subsequently, the images were converted into the format recommended by the Neuroimaging Informatics Technology Initiative (Nifti) to preserve the orientation information for further data processing. Then the files were compressed and the images were reoriented into right-anterior-inferior orientation with Convert3D.

Manual segmentations were performed in the portal-venous phase of contrast administration. This was performed for the baseline and follow-up CT studies. A semi-automated segmentation tool available in Amira Software (Thermo Fisher Scientific, Waltham, MA) was used to delineate the tumors, including the (i) enhancing portions, (ii) non-enhancing portions, and Lipiodol containing portions of the tumors. Enhancing tumor tissue was defined as a “region with uptake of contrast agent in the arterial phase of dynamic contrast CT” while non-enhancing tumor tissue was defined as a “region of no enhancement within HCC on the arterial phase images,” while Lipidol was defined as a “portions of high attenuation in pre-contrast images.” Manual segmentation of the whole tumor in the baseline study was done by one author (AM) to ensure the consistency of the segmentation throughout the dataset. The segmentation was done for all axial CT images. These manual segmentations provided the training data (*n* = 42 pairs) used to develop a neural network classifier for segmentation of tumors from the background liver tissue. Automated segmentation, performed using a CNN approach (U-Net), was used to segment the liver and tumor in two steps (24). To determine the correlation between mRECIST and MvRECIST and mRECIST and A-vRECIST, we compared the average uni-dimensional mRECIST measurements of the 3 readers to the M-vRECIST and A-vRECIST volumetric assessments.

### Statistical Analysis

Statistical analysis was performed using IBM SPSS Statistics V.24.0 software (IBM, Armonk, NY). Inter-reader reliability for mRECIST measurement was assessed using the intraclass correlation coefficient (ICC). The Pearson correlation coefficient (r) was used to measure the correlation between the diameter change (the average reading from mRECIST) and (i) the automated and (ii) manual volume changes after TACE. A *P*-value of 0.05 was used to determine the statistical significance of the measurements.

## Results

### Patient Characteristics

**Figure 2:**
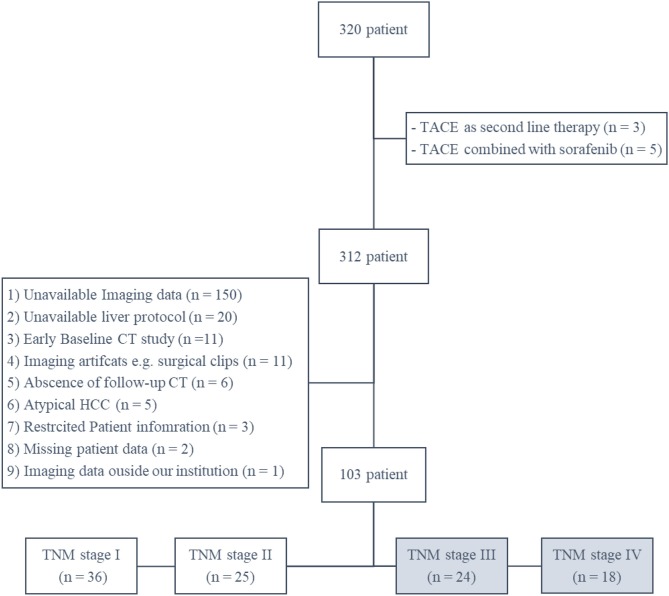
A schematic Flowchart of patient cohort selection process including the exclusion criteria.

There were 320 patients (with complete medical and survival data) found in our institutional database with HCC patients underwent TACE, we excluded 8 patients due to difference in their treatment plan (either TACE was used as second line or combined with other form of therapy). After thorough review of patients imaging studies, another 209 patients were excluded for different reasons (Figure 2). The final 103 patients were categorized according to their TNM stage into stage I, II, III, and IV with 36, 25, 24, and 18 patients. A total of 42 patients met our inclusion criteria for the study (TNM stage III and IV). On average, patients' baseline CT scans were performed 4 weeks (range between 2 and 7 weeks) before the first TACE session, and their follow-up scans were performed 11 weeks (range between 8 and 13 weeks) after the first TACE session. Patients' demographic data and baseline tumor characteristics are provided in Table 1.

**Table 1:** Patient demographic data and baseline tumor characteristics.

**Baseline characteristics**	**Value (*n* or mean ± SD) *n* = 42, 100%**
**Demographics**	
Age, years	67 ± 7
Sex ratio (male/female)	29 (69%)/13 (31%)
Cirrhosis (yes/no)	31 (74%)/11 (26%)
**Etiology**	
Tobacco use (yes/no)	28 (67%)/14 (33%)
Alcohol (yes/no)	25 (60%)/17 (40%)
Diabetes mellitus (yes/no)	12 (29%)/30 (71%)
Family history of cancer (yes/no)	24 (57%)/18 (43%)
Hepatitis (HBV/HCV/both/none)	3/7/8/24
**Tumor extension**	
Overall tumor size (cm)^*^	10 ± 5
Vascular invasion present	17 (40%)
Diffuse/infiltrative pattern	10 (24%)
Tumor involvement (≤50%/>50% of liver volume)	31/11
Alpha fetoprotein (ng/ml)^**^	38.5 ± 1567.3
Distant metastasis present	7 (17%)
Nodal metastasis present	14 (33%)
Portal vein thrombosis present	13 (31%)
Tumor nodularity (uni-/multilobular)	14/28
mRECIST category (CR/PR/SD/PD)	10/20/7/5
**HCC scoring**	
CLIP staging	
Stage 0	4 (9%)
Stage 1	17 (40%)
Stage 2	10 (24%)
Stage 3	7 (17%)
Stage 4	2 (5%)
Stage 5	2 (5%)
Okuda staging	
Stage I	26 (62%)
Stage II	16 (38%)
TNM staging	
Stage III	24 (57%)
Stage IV	18 (43%)
BCLC staging	
Stage B	7 (17%)
Stage C	35 (83%)

* *Overall tumor size was determined based on RECIST measurements*.

** *Alpha fetoprotein is reported using median ± interquartile range*.

*BCLC, Barcelona clinic liver cancer; CLIP, Cancer of the Liver Italian Program; HBV, hepatitis B virus; HCC, hepatocellular carcinoma; HCV, hepatitis C virus; RECIST, response evaluation criteria in solid tumors; SD, standard deviation; TNM, tumor-node-metastasis; CR, Complete Response; PR, Partial Response; SD, Stable disease; PD, Progressive Disease*.

### Manual and Automated Volumetric Assessment

The mRECIST response comparing the pre- and post-TACE images is listed in Table 2 for the three radiologists'.

**Table 2:** Radiologists' unidimensional mRECIST measurements of HCC tumors and average readings.

	**Baseline mRECIST (cm) Mean ± SD; 95% CI**	**Follow-up mRECIST (cm) Mean ± SD; 95% CI**	**Diameter change (cm)Mean ± SD; 95% CI**
Reader 1	7.9 ± 4.8 (6.4–9.4)	5.9 ± 4.7 (4.4–7.4)	−1.9 ± 4.1 (−3.3 to −0.7)
Reader 2	8.6 ± 5.3 (6.9–10.2)	6 ± 4.9 (4.4–7.5)	−2.6 ± 3.2 (−3.3 to −1.6)
Reader 3	7.8 ± 4.7 (6.4–9.4)	5.6 ± 4.9 (4.1–7.2)	−2.2 ± 2.9 (−3.2 to −1.3)
Average	8.1 ± 4.7 (6.6– 9.6)	5.9 ± 4.6 (4.4–7.3)	−2.3 ± 2.9 (−3.2 to −1.3)

*CI, confidence interval; HCC, hepatocellular carcinoma; mRECIST, modified response evaluation criteria in solid tumors; SD, standard deviation*.

The manual vRECIST and automated vRECIST response comparing the pre- and post TACE are presented in Table 3, A-vRECIST was obtained with CNN. Assessment with the ICC showed statistically significant correlation between the three readers (ICC = 0.824; 95% CI = 0.70–0.89; *P* < 0.001).

**Table 3:** Voxel-based volumetric measurements of HCC tumors made using M-vRECIST and A-vRECIST with CNN.

	**M-vRECIST Mean ± SD; 95% CI (cm^**3**^)**	**A-vRECIST with CNN Mean ± SD; 95% CI (cm^**3**^)**
Baseline study	466.8 ± 600 (279.8–653.9)	438.6 ± 552.9 (266.4–611)
Follow-up study	537.9 ± 772.8 (297.1–778.8)	426.2 ± 673.9 (216.3–636.3)
Volume change	71 ± 322.9 (−29.5–171.7)	−12.39 ± 395.4 (−135.6–110.8)

*A-vRECIST, automated volumetric response evaluation criteria in solid tumors; CI, confidence interval; CNN, convolutional neural network; HCC, hepatocellular carcinoma; M-vRECIST, manual volumetric response evaluation criteria in solid tumors; SD, standard deviation*.

To determine the correlation between mRECIST and vRECIST (both manual and automated), we compared the average unidimensional mRECIST measurements to the M-vRECIST and A-vRECIST volumetric assessments (Figure 1) and compared the diameter changes determined through the manual and automated volumetric assessments. The correlation between mRECIST and M-vRECIST was moderate for both the baseline and the follow-up studies (*r* = 0.622 and 0.748, respectively). The correlation between tumor diameter measurement changes was higher (*r* = 0.766). The differences between these measurements were statistically significant (*P* < 0.001 for all). The correlation between mRECIST and A-vRECIST was similar: *r* = 0.597 for the baseline and *r* = 0.648 for the follow-up studies (*P* < 0.001 for all). For the correlation between the baseline and follow-up tumor measurements, *r* = 0.774.

**Figure 3:**
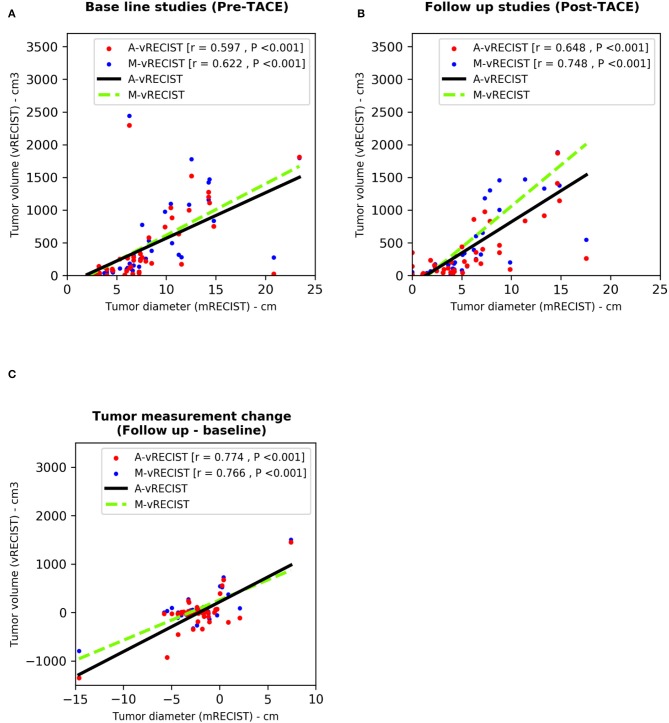
Scatter plots compare tumor assessments from unidimensional mRECIST with those from A-vRECIST and M-vRECIST in pre- and post-TACE studies [graphs **(A,B)**, respectively]. Graph **(C)** shows the difference between the A-vRECIST and M-vRECIST measurements. The best-fit lines and Pearson coefficients (r) are shown. A-vRECIST, automated volumetric response evaluation criteria in solid tumors; mRECIST, modified response evaluation criteria in solid tumors; M-vRECIST, manual volumetric response evaluation criteria in solid tumors; RECIST, response evaluation criteria in solid tumors; TACE, transarterial chemo embolization.

**Figure 4:**
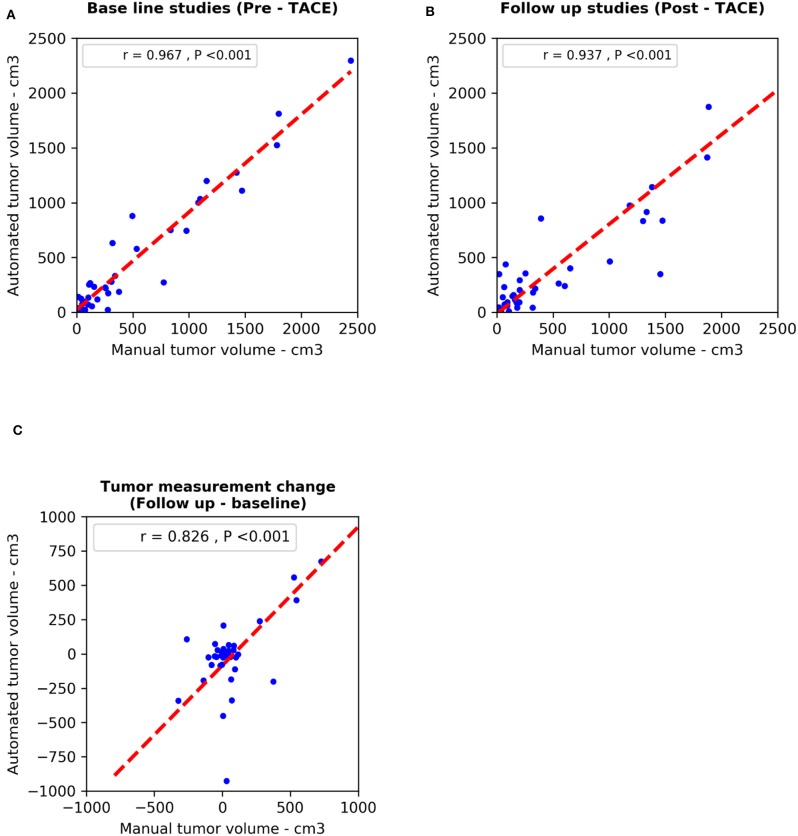
Scatter plots compare diameter changes from M-vRECIST vs. A-vRECIST assessments in the **(A)** baseline and **(B)** follow-up studies. Graph **(C)** shows the tumor measurement changes. The best-fit lines (dashed line) and Pearson correlation coefficients (r) are shown. A-vRECIST, automated volumetric response evaluation criteria in solid tumors; M-vRECIST, manual volumetric response evaluation criteria in solid tumors.

We used the Pearson correlation coefficient to compare M-vRECIST and A-vRECIST and found strong linear correlation between the two approaches (*r* = 0.967 for the baseline studies, *r* = 0.937 for the follow-up studies, and *r* = 0.826 for the tumor volume change after TACE [*P* < 0.001 for all]) (Figures 3, 4).

## Discussion

In this study, we hypothesized that, for large HCC tumors, assessment of volume changes before and after TACE using A-vRECIST would correlate with the measurement changes using uni-dimentional mRECIST and that, accordingly, A-vRECIST can be used to assess tumor response to TACE therapy.

Volumetric assessment of HCC have been emerged as recent tool for assessing of HCC response to treatment. Although assessment of treatment response of is some patterns of HCC is challenging (especially diffuse/infiltrative type) due to its indistinct borders. Previous studies showed that both uni-dimensional and volumetric measurement highly correlated with the actual pathological tumor volume, with volumetric assessment was similar to pathological volume while uni-dimensional measurement overestimate the volume by 28% (25, 26). Such studies demonstrate the superiority of volumetric assessment to estimate the real tumor volume, which is more important during assessment og treatment response.

Another study showed that HCC response to Sorafenib using volumetric assessment can be used an alternative tool for monitoring therapy better than mRECIST measurement (27). Another study used functional MRI volumetric analysis of HCC tumors to separate patients into responders and non-responders following treatment with combination TACE and Sorafenib (23, 28). Both of these studies made 3D measurements of HCC tumor volumes. However, manual volumetric assessment is time consuming and highly variable leading to motivate scientists to automate this process. Automated tumor volume and enhancement measurements using cross-sectional images are proven to be both reproducible and feasible in clinical application (29). In addition, it have been demonstrated that automated quantitative tumor volume assessment can become part of monitoring response to TACE (29).

There are challenges to the automated segmentation of the liver on CT. Among these is that the attenuation of adjacent organs and tissues that may be very similar to the liver tissue itself. In addition, model-based approaches to segmentation are challenging due to the liver's widely varying shape (30). Also, automated segmentation of small liver tumors showed lower accuracy compared to both manual segmentation and automated segmentation of larger tumors (12, 24). As a result, there have been multiple attempts to develop methods of automated liver segmentation using CT (31, 32).

In our study, we found that A-vRECIST measurements highly correlated with both M-vRECIST and unidimensional mRECIST measurements in large HCCs from patients who had undergone TACE.

Because mRECIST is currently the preferred method of monitoring TACE therapy, we used it as the standard to which vRECIST was compared. Our results showed that, between our experienced radiologists, there was moderate to high inter-reader agreement for monitoring therapy using mRECIST measurements (ICC = 0.824). Our study also showed that correlation between unidimensional mRECIST and the vRECIST measurements was good *r* = 0.766 for M-vRECIST and *r* = 0.774 for A-vRECIST.

**Figure 5:**
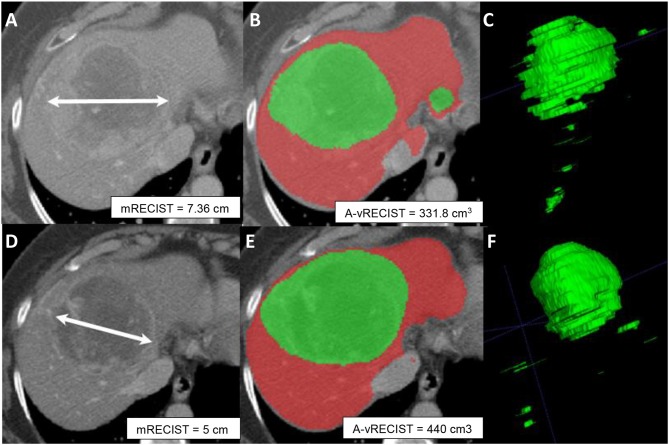
Axial sections from pre-TACE **(A–C)** and 4-week post-TACE **(D–F)** scans from a 70-year old male patient with advanced HCC. **(A,D)** Show changes in tumor size made with mRECIST measurements and indicative of tumor shrinkage. **(B,E)** Show A-vRECIST segmentation of the hepatic parenchyma (red) and the HCC tissue (green) and indicate tumor size increase. **(C,F)** Show the 3-dimensional voxel renderings of the A-vRECIST measurements.

Results of our vRECIST measurements are likely to provide an early marker for TACE monitoring (23, 28, 32). We found that A-vRECIST measurements made using our neural network model could be a good substitute for M-vRECIST measurements and mRECIST (Figure 5). It also can improve the workflow as an alternate measure of response assessment because the measurements were highly correlated to each other in the baseline study, follow-up study, and volume change results (*r* = 0.967, 0.937, and 0.826, respectively).

Our study had some limitations. First, the small cohort (42 patients) may have masked variability in the automated segmentation results. However, this study was a pilot, and we were aware from the outset that its findings would need to be confirmed prospectively in a larger population with more variable tumor sizes and stages. Second, we did not examine correlation between A-vRECIST and patient's outcome. However, this study serves as a step for further evaluation of clinical importance of A-vRECIST and its relation to patient's survival endpoints. The small differences observed between A-vRECIST and M-vRECIST in the follow-up images (*r* = 0.648 vs. 0.748, respectively) may have been due to differences in TACE techniques, as the Lipiodol used in conventional TACE can distort CNNs, leading to differences in automated tumor segmentation.

Our next step is to confirm our findings with a larger sample size offering higher variability in tumor sizes and stages. We plan to use A-vRECIST results to classify patients according to their responses to TACE (partial response vs. no response). Also, we will thoroughly study the confounding factors, such as chronic parenchymal liver disease, that may affect the performance of neural networks.

## Data Availability Statement

The datasets generated for this study are available on request to the corresponding author.

## Ethics Statement

The study was approved by the Institutional Review Board (IRB) at MD Anderson Cancer Center, office of protocol research. The informed consent was waived and approved by our institutional IRB.

## Author Contributions

AM contributed to the planning of the project, data collection, curation, data analysis, preparing figures, and write the first draft of the manuscript. KB helped in data curation, planning of the project, and reviewed the final draft of manuscript. AK contributed to the planning of the project, data collection, and shared in writing the first draft of the manuscript. DF and JH technical leaders of the project, construction of the neural network we used, planning of the project, and reviewing the first draft of the project. JS, AQ, and KE three radiologists who read the RECIST of the tumors, helped in planning the project, and also reviewed the final draft of the manuscript. KE provided supervision, support, conceptualization, and guidance throughout the project.

## Conflict of Interest

The authors declare that the research was conducted in the absence of any commercial or financial relationships that could be construed as a potential conflict of interest.

## Abbreviations

A-vRECIST: automated volumetric response evaluation criteria in solid tumors
CNN: convolutional neural network
CT: computed tomography
HCC: hepatocellular carcinoma
ICC: intra-class correlation coefficient
mRECIST: modified response evaluation criteria in solid tumors
MRI: magnetic resonance imaging
M-vRECIST: manual volumetric response evaluation criteria in solid tumors
RECIST: response evaluation criteria in solid tumors
TACE: transarterial chemo embolization
TNM: tumor-node-metastasis
vRECIST: volumetric response evaluation criteria in solid tumors.
